# Supplementary material for: Engineered E. coli Nissle 1917 for the delivery of matrix-tethered therapeutic domains to the gut
Source: Nat Commun. 2019 Dec 6;10:5580. doi: 10.1038/s41467-019-13336-6 (PMC6898321; doi:10.1038/s41467-019-13336-6)
Supplement: Supplementary file 1 — Supplementary Information [file 41467_2019_13336_MOESM1_ESM.pdf]

Supplementary Information

Engineered *E. coli* Nissle 1917 for the delivery of matrix-tethered therapeutic domains to the gut

Authors:

Pichet Praveschotinunt[1,2], Anna M. Duraj-Thatte[1,2], Ilia Gelfat,[1,2], Franziska Bahl,[1,3], David B. Chou[1,4], Neel S. Joshi[1,2,*]

Affiliations:

[1] Wyss Institute for Biologically Inspired Engineering, Harvard University, Boston, MA, United States

[2] John A. Paulson School of Engineering and Applied Sciences, Harvard University, Cambridge, MA, United States

[3] Faculty of Biology, Albert Ludwigs University of Freiburg, Freiburg im Breisgau, Germany

[4] Department of Pathology, Massachusetts General Hospital, Boston, MA

*Corresponding Author. Contact email: neel.joshi@wyss.harvard.edu

Supplementary figures

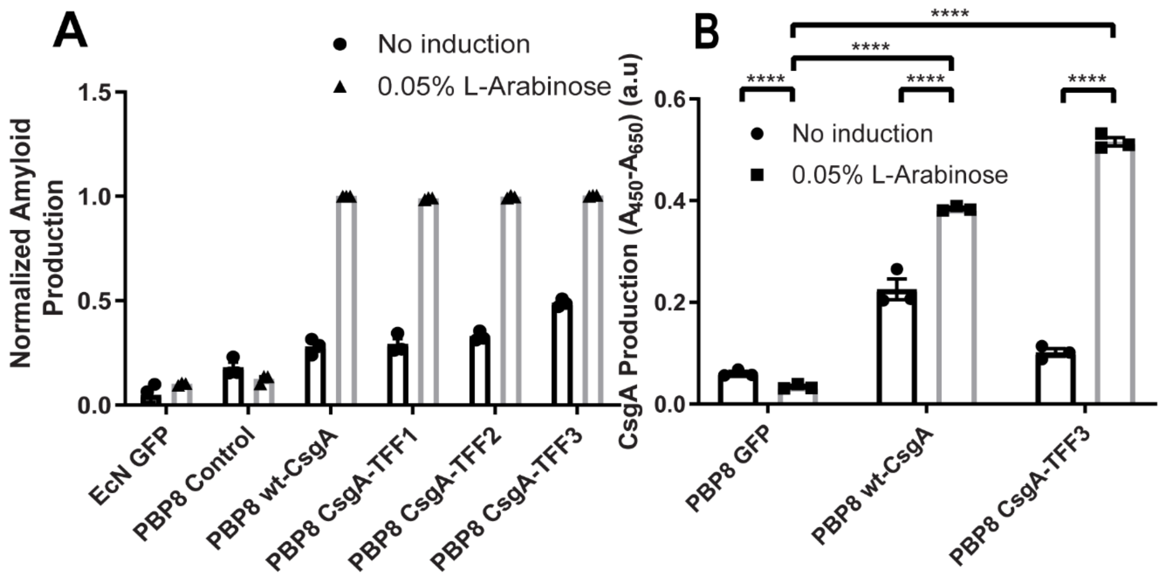

Supplementary Figure 1. (A) Normalized amyloid production of induced and non-induced PBP8 library. (B) Relative CsgA production of induced and non-induced PBP8 library. Data are represented as mean ± SEM. See materials and methods for statistics.

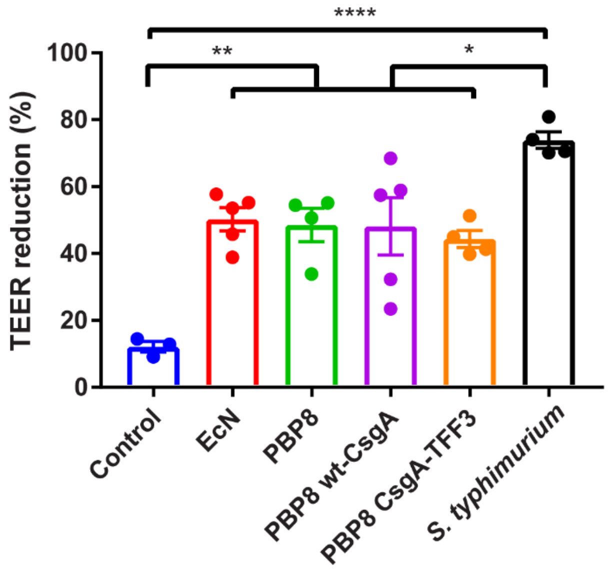

Supplementary Figure 2. Changes in the trans-epithelial electrical resistance (TEER) of polarized Caco-2 cells at 24 hours after infection, and the percentage of reduction was calculated. Data are represented as mean ± SEM. See materials and methods for statistics.

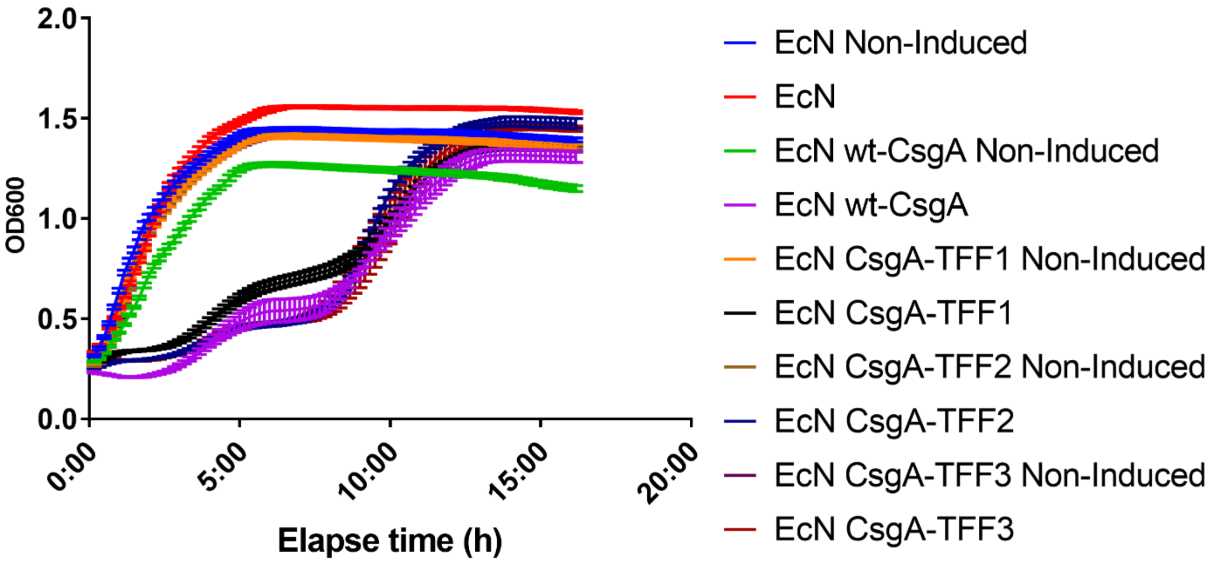

Supplementary Figure 3. *In vitro* growth curve of EcN Prop-luc library (N=6). Data are represented as mean ± SEM. See materials and methods for statistics.

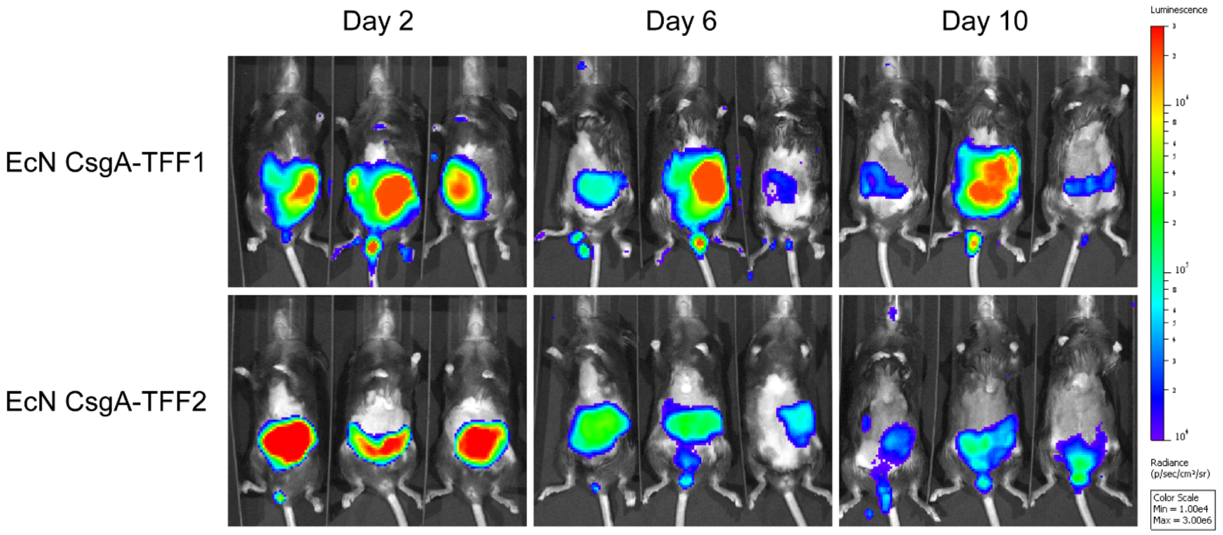

Supplementary Figure 4. IVIS images of mice received EcN Prop-luc CsgA-TFF1 or EcN CsgA-TFF2. Images were taken at day 2, 6 and 10 post-inoculated.

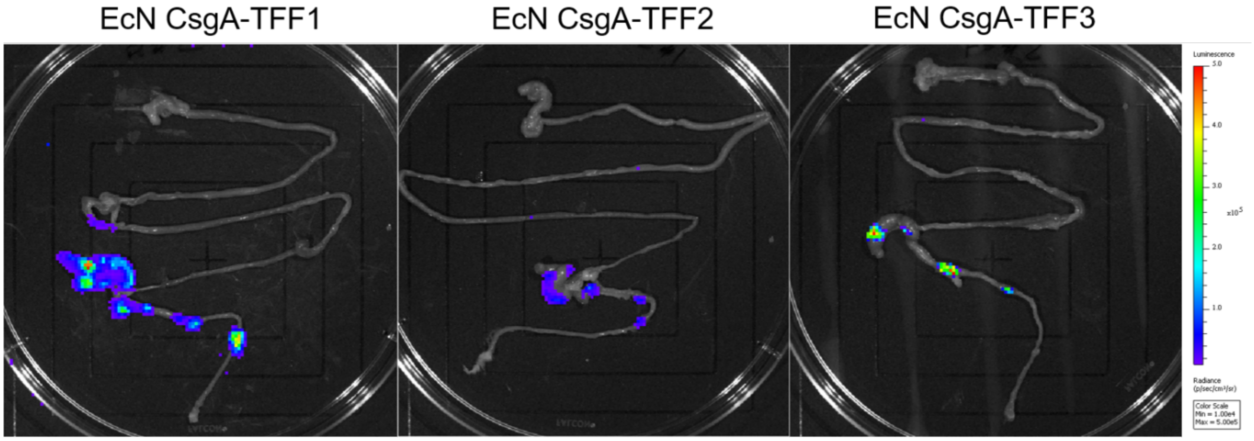

Supplementary Figure 5. *Ex vivo* IVIS images of mouse guts received EcN Prop-luc CsgA-TFF1, EcN CsgA-TFF2, and EcN CsgA-TFF3 showing localization of the engineered EcN. Images were taken at day 2 post-inoculated.

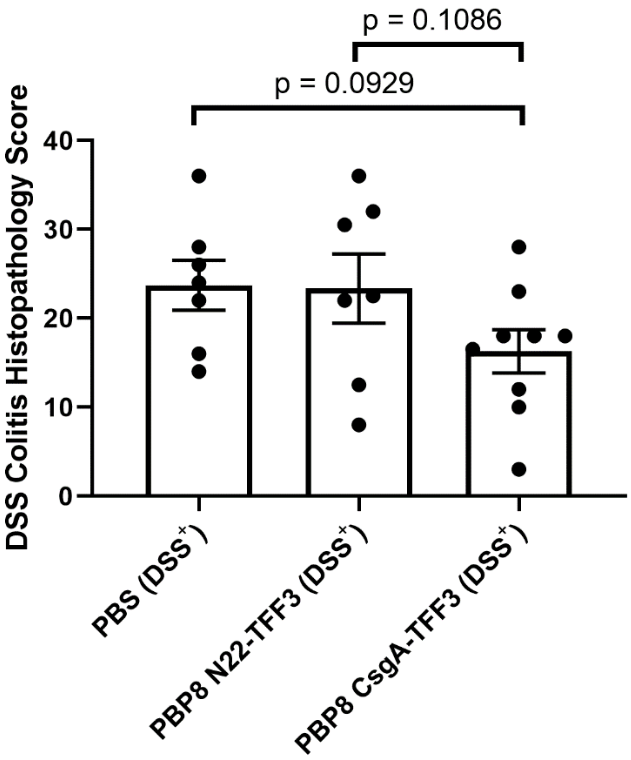

Supplementary Figure 6. Combined DSS colitis histopathology score reflecting severity of inflammation (see Table S1 for details) for three conditions: PBS (DSS+), PBP8 N22-TFF3 (DSS+), and PBP8 CsgA-TFF3 (DSS+) (N = 6-8).

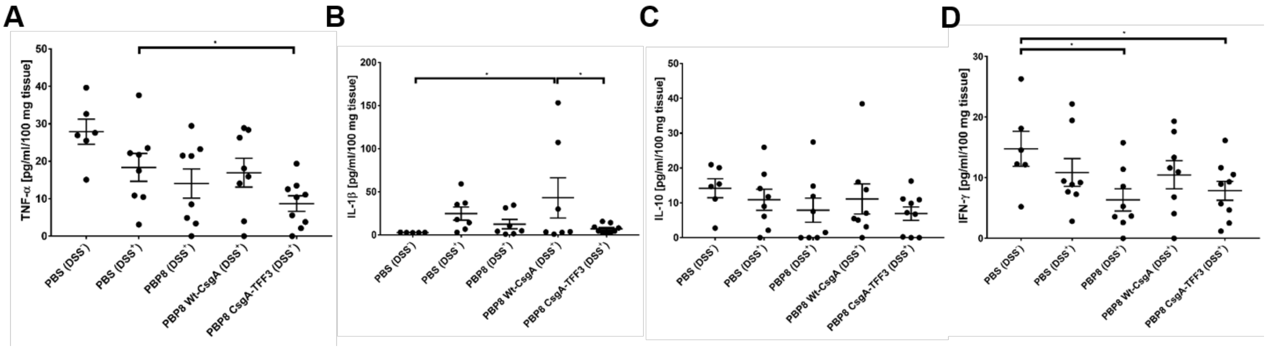

Supplementary Figure 7. Effects of engineered EcN treatment on colonic cytokine expression in DSS-induced colitic mice. (A-D) TNF-α, IL-1β, IL10, IFN-γ protein level determined by multiplex ELISA from homogenized distal colon of mice. Data are presented as protein concentration per 100 mg of tissue from two independent experiments (N=6-9). Data are represented as mean ± SEM. See materials and methods for statistics.

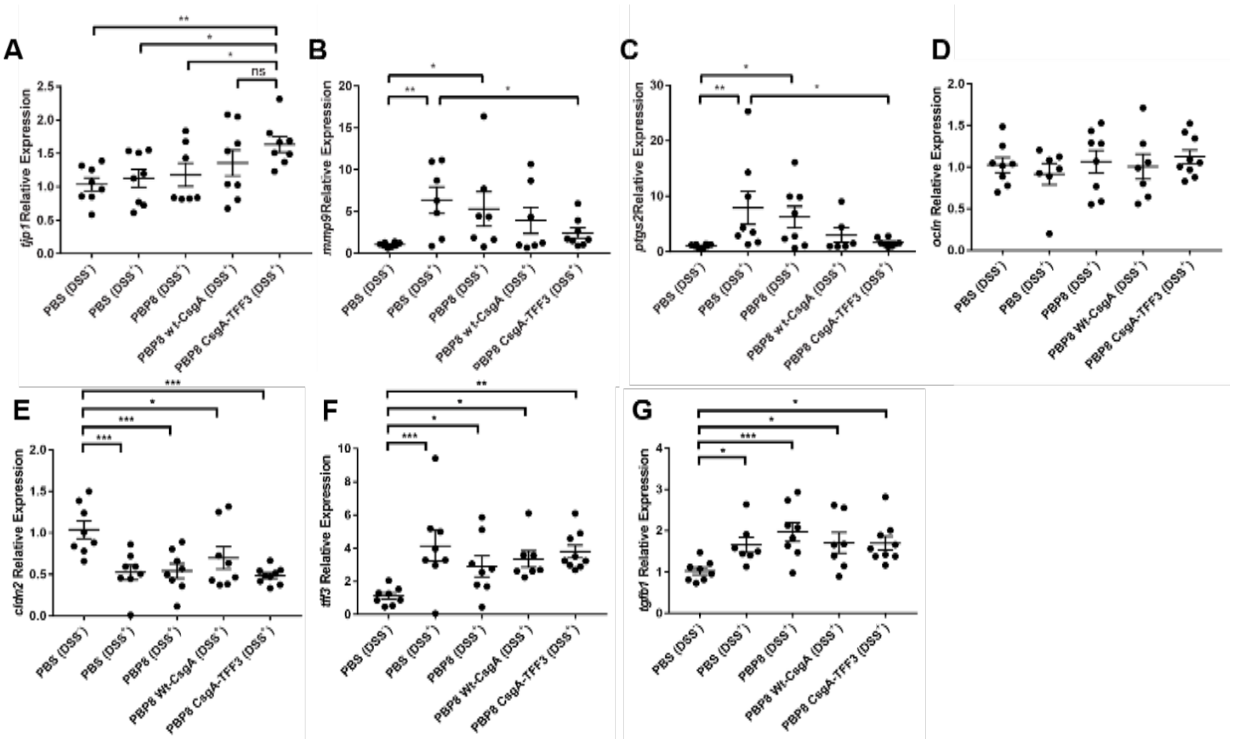

Supplementary Figure 8. Effects of engineered EcN treatment on colonic gene expression in DSS-induced colitic mice (A-G) *tjp1, mmp9, ptgs2, ocln, cldn2, tff3* and *tgfb1* expression measured by qRT-PCR from homogenized distal colon of mice. Data are presented as fold change compared to the healthy control group from two independent experiments (N=7-9). Data are represented as mean ± SEM. See materials and methods for statistics.

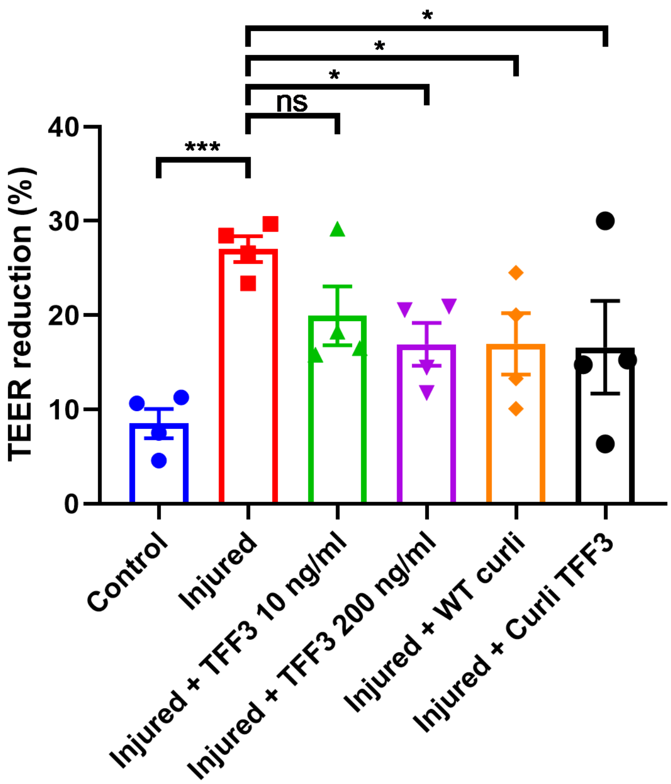

Supplementary Figure 9. Changes in the trans-epithelial electrical resistance (TEER) of polarized Caco-2 cells at 24 hours after incubation with 10 ng/ml interferon-γ (IFN-γ) and 10 ng/ml tumor necrosis factor-α (TNF-α) and various experimental conditions (Control = no IFN-γ and TNF-α, Injured = with IFN-γ and TNF-α). The percentage of reduction was calculated normalized to pre-incubation time point. Data are represented as mean ± SEM (N = 4). See materials and methods for statistics.

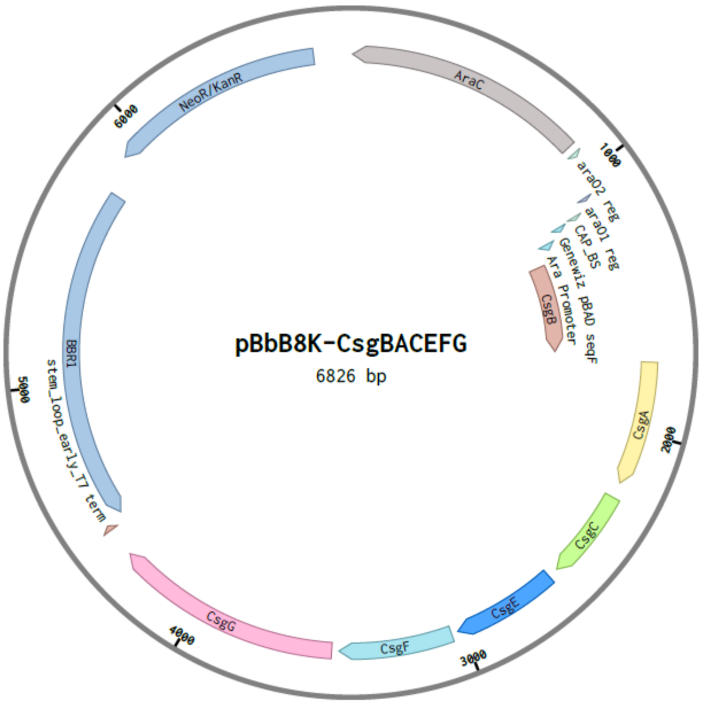

Supplementary Figure 10. pBbB8k-wt-CsgA plasmid map.

Supplementary tables

Supplementary Table 1 CsgA fusion DNA sequences

Plasmid name	CsgA Fusion DNA Sequences
pBbB8k-wt-CsgA	ATGAAACTTTTAAAAGTAGCAGCAATTGCAGCAATCGTATT<br>CTCCGGTAGCGCTCTGGCAGGTGTTGTTCCTCAGTACGGCG<br>GCGGCGGTAACCACGGTGGTGGCGGTAATAATAGCGGCCC<br>AAATTCTGAGCTGAACATTTACCAGTACGGTGGCGGTAACT<br>CTGCACTTGCTCTGCAAACTGATGCCCGTAACTCTGACTTG<br>ACTATTACCCAGCATGGCGGCGGTAATGGTGCAGATGTTG<br>GTCAGGGCTCAGATGACAGCTCAATCGATCTGACCCAACG<br>TGGCTTCGGTAACAGCGCTACTCTTGATCAGTGGAACGGCA<br>AAAATTCTGAAATGACGGTTAAACAGTTCGGTGGTGGCAA<br>CGGTGCTGCAGTTGACCAGACTGCATCTAACTCCTCCGTCA<br>ACGTGACTCAGGTTGGCTTTGGTAACAACGCGACCGCTCAT<br>CAGTACGGCTCTGGTGGCTCTGGTGGCTCTGGCGGCAGCG<br>GGCATCACCACCACCATCATTAA
pBbB8k-CsgA-TFF1	ATGAAACTTTTAAAAGTAGCAGCAATTGCAGCAATCGTATT<br>CTCCGGTAGCGCTCTGGCAGGTGTTGTTCCTCAGTACGGCG<br>GCGGCGGTAACCACGGTGGTGGCGGTAATAATAGCGGCCC<br>AAATTCTGAGCTGAACATTTACCAGTACGGTGGCGGTAACT<br>CTGCACTTGCTCTGCAAACTGATGCCCGTAACTCTGACTTG<br>ACTATTACCCAGCATGGCGGCGGTAATGGTGCAGATGTTG<br>GTCAGGGCTCAGATGACAGCTCAATCGATCTGACCCAACG<br>TGGCTTCGGTAACAGCGCTACTCTTGATCAGTGGAACGGCA<br>AAAATTCTGAAATGACGGTTAAACAGTTCGGTGGTGGCAA<br>CGGTGCTGCAGTTGACCAGACTGCATCTAACTCCTCCGTCA<br>ACGTGACTCAGGTTGGCTTTGGTAACAACGCGACCGCTCAT<br>CAGTACGGTGGAGGTTCCGGGGGCGGTTCTGGCGGCGGAA<br>GTGGGGGTGGCTCGGGTGGGGGATCAGGTGGAGGCAGCGG<br>AGGTGGATCGGGGGGAGGGAGTGGTGGCGGTTCGGGTGGT<br>GGGTCAGGGGGAGGGTCGGGCGGGGGTAGTCATCATCATC<br>ATCACCATGGGGGAGGTTCGGGCGGAGGATCGGGGGGAG<br>GCAGCGAAGCACAAACAGAGACCTGCACCGTTGCACCACG<br>CGAACGTCAAAATTGCGGGTTTCCAGGGGTGACGCCATCT<br>CAGTGCGCCAATAAGGGGTGCTGCTTCGACGACACTGTTC<br>GCGGCGTTCCTTGGTGCTTCTATCCGAACACTATCGATGTA<br>CCTCCTGAAGAAGAATGCGAATTTTAA

pBbB8k-CsgA-TFF2	ATGAAACTTTTAAAAGTAGCAGCAATTGCAGCAATCGTATT<br>CTCCGGTAGCGCTCTGGCAGGTGTTGTTCCTCAGTACGGCG<br>GCGGCGGTAACCACGGTGGTGGCGGTAATAATAGCGGCCC<br>AAATTCTGAGCTGAACATTTACCAGTACGGTGGCGGTAACT<br>CTGCACTTGCTCTGCAAACTGATGCCCGTAACTCTGACTTG<br>ACTATTACCCAGCATGGCGGCGGTAATGGTGCAGATGTTG<br>GTCAGGGCTCAGATGACAGCTCAATCGATCTGACCCAACG<br>TGGCTTCGGTAACAGCGCTACTCTTGATCAGTGGAACGGCA<br>AAAATTCTGAAATGACGGTTAAACAGTTCGGTGGTGGCAA<br>CGGTGCTGCAGTTGACCAGACTGCATCTAACTCCTCCGTCA<br>ACGTGACTCAGGTTGGCTTTGGTAACAACGCGACCGCTCAT<br>CAGTACGGTGGAGGGAGCGGAGGGGGTAGTGGAGGAGGC<br>TCTGGGGGGGGCAGTGGTGGCGGCTCCGGTGGTGGGTCTG<br>GAGGTGGAAGTGGGGGAGGGTCGGGCGGCGGCTCGGGTG<br>GGGGATCTGGTGGTGGGAGTGGGGGGGGTAGTCATCATCA<br>CCACCATCATGGGGGAGGGAGCGGTGGCGGCTCTGGAGGG<br>GGGTCCGAAAAGCCATCCCCGTGCCAGTGTTCTCGTTTGTC<br>GCCCCACAATCGCACTAATTGTGGGTTTCCCGGCATCACGT<br>CAGACCAATGCTTTGACAACGGATGCTGCTTCGACTCGTCC<br>GTTACGGGAGTCCCCTGGTGTTTTCACCCGCTGCCTAAGCA<br>AGAGTCTGACCAATGCGTTATGGAGGTAAGCGATCGCCGT<br>AATTGTGGTTATCCGGGTATCAGTCCTGAAGAATGCGCATC<br>CCGCAAATGCTGCTTTAGCAACTTCATTTTCGAAGTACCCT<br>GGTGTTTCTTCCCTAAGTCAGTAGAGGATTGTCACTATTAA
pBbB8k-CsgA-TFF3	ATGAAACTTTTAAAAGTAGCAGCAATTGCAGCAATCGTATT<br>CTCCGGTAGCGCTCTGGCAGGTGTTGTTCCTCAGTACGGCG<br>GCGGCGGTAACCACGGTGGTGGCGGTAATAATAGCGGCCC<br>AAATTCTGAGCTGAACATTTACCAGTACGGTGGCGGTAACT<br>CTGCACTTGCTCTGCAAACTGATGCCCGTAACTCTGACTTG<br>ACTATTACCCAGCATGGCGGCGGTAATGGTGCAGATGTTG<br>GTCAGGGCTCAGATGACAGCTCAATCGATCTGACCCAACG<br>TGGCTTCGGTAACAGCGCTACTCTTGATCAGTGGAACGGCA<br>AAAATTCTGAAATGACGGTTAAACAGTTCGGTGGTGGCAA<br>CGGTGCTGCAGTTGACCAGACTGCATCTAACTCCTCCGTCA<br>ACGTGACTCAGGTTGGCTTTGGTAACAACGCGACCGCTCAT<br>CAGTACGGTGGTGGTAGTGGTGGCGGCAGTGGTGGCGGTA<br>GCGGCGGTGGCTCCGGTGGCGGTTCTGGCGGCGGTTCTGGT<br>GGTGGTTCTGGCGGTGGCTCAGGTGGGGGTTCCGGCGGCG<br>GTAGCGGCGGTGGATCTGGCGGCGGCTCTCATCATCATCAT<br>CATCATGGTGGTGGTTCTGGCGGTGGCTCCGGTGGTGGCTC<br>TGAAGAATATGTGGGCCTGAGCGCGAACCAGTGCGCGGTG<br>CCGGCGAAAGATCGCGTGGATTGCGGCTATCCGCATGTGA<br>CCCCGAAAGAATGCAACAACCGCGGCTGCTGCTTTGATAG<br>CCGCATTCCGGGCGTGCCGTGGTGCTTTAAACCGCTGCAGG<br>AAGCGGAATGCACCTTTTAA

pBbB8k-N22-TFF3	GGTGTTGTTCCTCAGTACGGCGGCGGCGGTAACCACGGTG GTGGCGGTAATAATAGCGGCCCAAATGGGGGAGGATCAGG AGGCGGCTCAGGTGGAGGCAGTCATCATCATCATCACCAT GGAGGAGGATCAGGTGGCGGATCCGGCGGTGGATCCGAAG AGTACGTGGGCCTGAGTGCAAATCAATGCGCCGTGCCCGC TAAAGATCGTGTCGATTGCGGGTACCCTCATGTTACCCCAA AGGAGTGTAATAATCGCGGTTGCTGTTTTGATTCGCGTATT CCTGGGGTGCCTTGGTGTTTAAACCCTTGCAAGAAGCAGA ATGCACCTTTTAA

Supplementary Table 2 CsgA fusion amino acid sequences

Plasmid name	CsgA Fusion Amino Acid Sequences
pBbB8k-wt-CsgA	MKLLKVAAIAAIVFSGSALAGVVPQYGGGGNHGGGGNNSGPNSELN IYQYGGGNSALALQTDARNSDLTITQHGGGNGADVGQGSDDSSIDLT QRGFGNSATLDQWNGKNSEMTVKQFGGGNGAAVDQTASNSSVNVT QVGFGNNATAHQYGSGGSGGSGGSGHHHHHH*
pBbB8k-CsgA-TFF1	MKLLKVAAIAAIVFSGSALAGVVPQYGGGGNHGGGGNNSGPNSELN IYQYGGGNSALALQTDARNSDLTITQHGGGNGADVGQGSDDSSIDLT QRGFGNSATLDQWNGKNSEMTVKQFGGGNGAAVDQTASNSSVNVT QVGFGNNATAHQYGGGSGGGSGGGSGGGSGGGSGGGSGGGSGGGS GGGSGGGSGGGSGGGSHHHHHHGGGSGGGSGGGSEAQTETCTVAP RERQNCGFPGVTPSQCANKGCCFDDTVRGVPWCFYPNTIDVPPEEEC EF*
pBbB8k-CsgA-TFF2	MKLLKVAAIAAIVFSGSALAGVVPQYGGGGNHGGGGNNSGPNSELN IYQYGGGNSALALQTDARNSDLTITQHGGGNGADVGQGSDDSSIDLT QRGFGNSATLDQWNGKNSEMTVKQFGGGNGAAVDQTASNSSVNVT QVGFGNNATAHQYGGGSGGGSGGGSGGGSGGGSGGGSGGGSGGGS GGGSGGGSGGGSGGGSHHHHHHGGGSGGGSGGGSEKPSPCQCSRLS PHNRTNCGFPGITSDQCFDNGCCFDSSVTGVPWCFHPLPKQESDQCV MEVSDRRNCGYPGISPEECASRKCCFSNFIFEVPWCFFPKSVEDCHY*
pBbB8k-CsgA-TFF3	MKLLKVAAIAAIVFSGSALAGVVPQYGGGGNHGGGGNNSGPNSELN IYQYGGGNSALALQTDARNSDLTITQHGGGNGADVGQGSDDSSIDLT QRGFGNSATLDQWNGKNSEMTVKQFGGGNGAAVDQTASNSSVNVT QVGFGNNATAHQYGGGSGGGSGGGSGGGSGGGSGGGSGGGSGGGS GGGSGGGSGGGSGGGSHHHHHHGGGSGGGSGGGSEEYVGLSANQC AVPAKDRVDCGYPHVTPKECNNRGCCFDSRIPGVPWCFKPLQEAECT F*
pBbB8k-N22-TFF3	GVVPQYGGGGNHGGGGNNSGPNGGGSGGGSGGGSHHHHHHGGGS GGGSGGGSEEYVGLSANQCAVPAKDRVDCGYPHVTPKECNNRGCCF DSRIPGVPWCFKPLQEAECTF

Supplementary Table 3 Cloning and sequencing primers

Primer Name	Sequence	Reference
Forward primer to open pBbB8k at the end of CsgA	5'TAATACATCATTTGTATTACAGAAACAGGG CGC	This study
Reverse primer to open pBbB8k at the end of CsgA	5'GTACTGATGAGCGGTCGCGTTGTT	This study
Forward sequencing primer for CsgA fusion	5'GGTAATACTGCGATGATTATCCAG	This study
Reverse sequencing primer for CsgA fusion	5'CACAGACAAGATTGAGTAAGAGTG	This study
Forward primer to open pBbB8k at the end of N22 sequence in CsgA	5'ATTTGGGCCGCTATTATTACCGCC	This study

Supplementary Table 4 Bacterial strains and plasmids

Designation	Genotype	Reference
Bacterial strains		
E. coli Nissle 1917 (EcN)	Human commensal *E. coli* isolate	[1]
Prop-luc	EcN *luxABCDE erm*(Erythromycin Resistance)	[2]
PBP8	EcN Δcsg operon::CAT (Choloramphenicol Resistance)	[3]
Plasmids		
pBbB8k-GFP	GFP expressing, arabinose inducible plasmid, kanamycin resistance	Addgene#35363
pBbB8k-wt-CsgA	wt-CsgA expressing, arabinose inducible plasmid, kanamycin resistance	[3]
pBbB8k-CsgA-TFF1	CsgA-TFF1 expressing, arabinose inducible plasmid, kanamycin resistance	This study
pBbB8k-CsgA-TFF2	CsgA-TFF2 expressing, arabinose inducible plasmid, kanamycin resistance	This study
pBbB8k-CsgA-TFF3	CsgA-TFF3 expressing, arabinose inducible plasmid, kanamycin resistance	This study
pBbB8k- N22-TFF3	N22-TFF3 expressing, arabinose inducible plasmid, kanamycin resistance	This study

Supplementary Table 5 qRT-PCR primer set

Organism	Target genes	Protein	Sequence	Reference	Tm	Accesion number
Mus musculus	*gapdh*	Gapdh	5'-CTTTGTCAAGCTCATTTCCTGG-3'	This study	61.1	NM_008084
			5'-TCTTGCTCAGTGTCCTTGC-3'		57.6	
Mus musculus	*ocln*	Occludin	5'-ACTATGCGGAAAGAGTTGACAG-3'	This study	58.5	NM_008756
			5'-GTCATCCACACTCAAGGTCAG-3'		58.1	
Mus musculus	*tgfb1*	TGF-β1	5'-CCTGAGTGGCTGTCTTTTGA-3'	This study	59	NM_011577
			5'-CGTGGAGTTTGTTATCTTTGCTG-3'		61	
Mus musculus	*mmp9*	MMP-9	5'-GATCCCCAGAGCGTCATTC-3'	This study	60.6	NM_013599
			5'-CCACCTTGTTCACCTCATTTTG-3'		61.7	
Mus musculus	*ptgs2*	COX2	5'-CTCACGAAGGAACTCAGCAC-3'	This study	58.6	NM_011198
			5'-GGATTGGAACAGCAAGGATTTG-3'		62.9	
Mus musculus	*tff3*	TFF3	5'-CTGGGATAGCTGCAGATTACG-3'	This study	59.9	NM_011575
			5'-AGGGCACATTTGGGATACTG-3'		59.8	
Mus musculus	*tjp1*	ZO-1	5'-AGCGAATGTCTAAACCTGGG-3'	This study	59.2	NM_009386
			5'-TCCAACTTGAGCATACACAGG-3'		58.8	

Mus musculus	*cldn2*	Claudin-2	5’-CCAAACGACAAGCAAACAGG-3’	This study	61.6	NM_016675
			5’-CAGCATGGCAATGGATGTG-3’		62.1	

Supplementary Reagent List

Reagents and instruments (in order of appearance)	Distributors
Congo red	Sigma
50X Phosphate buffered saline (PBS)	Teknova
96 well clear bottom plate	Corning
Biotek H1 Microplate Reader	Biotek
20X Tris-buffered saline (TBS)	Sigma
20X Tris-buffered saline with 0.1% tween-20 (TBST)	Sigma
Bovine serum albumin (BSA)	Sigma
Hydrogen Peroxide	Sigma
Anti-6xHis antibody-horseradish peroxidase (HRP) (MA1-80218)	Thermo Fisher Scientific
Anti-TFF3 primary antibody (WH0007033M1)	Sigma
Goat anti-mouse-HRP conjugated secondary antibody (31430)	Thermo Fisher Scientific
Ultra-TMB (3,3',5,5'-tetramethylbenzidine) ELISA substrate	Thermo Fisher Scientific
6M Sulfuric acid	Alfa Aesar
Nucleopore Track-Etched membranes (0.22 μm pore size)	GE Healthcare Biosciences
4% Glutaldehyde in 0.1 M Sodium cacodylate buffer	Electron Microscopy Sciences
16% Paraformaldehyde	Electron Microscopy Sciences
0.4 M Sodium cacodylate buffer	Electron Microscopy Sciences
Autosamdri-931 critical point dryer	Tousimis
Carbon adhesives	Electron Microscopy Sciences
24-well plate, tissue culture treated	Falcon
DMEM with 1 g/L glucose	Gibco
Fetal bovine serum (FBS)	Gibco
Gentamicin	Sigma
Triton-X	Sigma
Millicell ERS-2 Voltohmmeter	Millipore
FITC-dextran	Sigma
96-well plate, tissue culture treated	Falcon
Human IL-8/CXCL8 Duo Set ELISA, R&D Systems	R&D System
L-(+)-arabinose	Sigma
Kanamycin	RPI
IVIS Lumina II	PerkinElmer
Multiscreen-GV 96-well filter plate	Millipore

RNAlater solution	Sigma
5X mammalian cell lysis buffer	Abcam
5 mm-stainless steel beads	Qiagen
TissueLyser LT	Qiagen
Bio-Plex Pro Mouse Cytokine Th17 Panel A 6-Plex kit	Bio-Rad
BioPlex 3D system	Bio-Rad
RNeasy plus mini kit	Qiagen
Nanodrop 2000c	Thermo Fisher Scientific
KAPA SYBR FAST One-Step qRT-PCR kit	Kapa Biosystems
CFX96 real time PCR detection system	Bio-Rad

Supplementary References

1. Sonnenborn, U. & Schulze, J. The non-pathogenicEscherichia colistrain Nissle 1917 – features of a versatile probiotic. *Microbial Ecology in Health and Disease.* **21**, 122-158 (2009).
2. Danino, T. *et al. Programmable probiotics for detection of cancer in urine*. Vol. 7 (2015).
3. Praveschotinunt, P. *et al.* Tracking of Engineered Bacteria In Vivo Using Nonstandard Amino Acid Incorporation. *ACS Synthetic Biology.* **7**, 1640-1650 (2018).
