# Supplementary material for: Engineered E. coli Nissle 1917 for the delivery of matrix-tethered therapeutic domains to the gut
Source: Nat Commun. 2019 Dec 6;10:5580. doi: 10.1038/s41467-019-13336-6 (PMC6898321; doi:10.1038/s41467-019-13336-6)
Supplement: Supplementary file 3 — Reporting Summary [file 41467_2019_13336_MOESM3_ESM.pdf]

nature research

Corresponding author(s): NCOMMS-18-28827A

Last updated by author(s): May 16, 2019

Reporting Summary

Nature Research wishes to improve the reproducibility of the work that we publish. This form provides structure for consistency and transparency in reporting. For further information on Nature Research policies, see Authors & Referees and the Editorial Policy Checklist.

Statistics

For all statistical analyses, confirm that the following items are present in the figure legend, table legend, main text, or Methods section.

n/a	Confirmed	
☐	☒	The exact sample size (*n*) for each experimental group/condition, given as a discrete number and unit of measurement
☐	☒	A statement on whether measurements were taken from distinct samples or whether the same sample was measured repeatedly
☐	☒	The statistical test(s) used AND whether they are one- or two-sided<br>*Only common tests should be described solely by name; describe more complex techniques in the Methods section.*
☐	☒	A description of all covariates tested
☐	☒	A description of any assumptions or corrections, such as tests of normality and adjustment for multiple comparisons
☐	☒	A full description of the statistical parameters including central tendency (e.g. means) or other basic estimates (e.g. regression coefficient) AND variation (e.g. standard deviation) or associated estimates of uncertainty (e.g. confidence intervals)
☐	☒	For null hypothesis testing, the test statistic (e.g. F, t, r) with confidence intervals, effect sizes, degrees of freedom and P value noted<br>*Give P values as exact values whenever suitable.*
☒	☐	For Bayesian analysis, information on the choice of priors and Markov chain Monte Carlo settings
☒	☐	For hierarchical and complex designs, identification of the appropriate level for tests and full reporting of outcomes
☒	☐	Estimates of effect sizes (e.g. Cohen's *d*, Pearson's *r*), indicating how they were calculated

Our web collection on statistics for biologists contains articles on many of the points above.

Software and code

Policy information about availability of computer code

Data collection	Microsoft Excel, Living Image Software 4, BioTek Gen5
Data analysis	GraphPad Prism 8

For manuscripts utilizing custom algorithms or software that are central to the research but not yet described in published literature, software must be made available to editors/reviewers. We strongly encourage code deposition in a community repository (e.g. GitHub). See the Nature Research guidelines for submitting code & software for further information.

Data

Policy information about availability of data

All manuscripts must include a data availability statement. This statement should provide the following information, where applicable:

- Accession codes, unique identifiers, or web links for publicly available datasets
- A list of figures that have associated raw data
- A description of any restrictions on data availability

The authors declare that all relevant data supporting the findings of this study are available within the article and its Supplementary Information Files or from the corresponding author on request.

Field-specific reporting

Please select the one below that is the best fit for your research. If you are not sure, read the appropriate sections before making your selection.

☒ Life sciences ☐ Behavioural & social sciences ☐ Ecological, evolutionary & environmental sciences

For a reference copy of the document with all sections, see nature.com/documents/nr-reporting-summary-flat.pdf

Life sciences study design

All studies must disclose on these points even when the disclosure is negative.

Sample size	Normally, in the in vitro bacterial experiments Congo Red and whole cell ELISA, we use at least three separate colonies of bacteria as a sample size. In cell culture experiment, we used at least four samples from two independent experiments. In mouse experiment, we used around 6-10 samples from two independent experiments. We did not perform power calculation prior to the experiments, but we think that the number of samples are adequate and yield significant difference in terms of statistics.
Data exclusions	Most of the data were not excluded. In certain mouse experiments, the data were excluded based on outlier removal mechanisms in GraphPad Prism 8 with ROUT techniques (Q=1%)
Replication	Tissue culture and mouse experiments were performed twice independently to confirm the result of the experiments.
Randomization	Bacterial colonies were picked at random. Mice were assorted randomly to cages when received from the vendor.
Blinding	Blinding was not possible since the experimenter was the one who prepared the samples and tested them in different models. Most of the experimental data were quantitative, so blinding was not necessary.

Reporting for specific materials, systems and methods

We require information from authors about some types of materials, experimental systems and methods used in many studies. Here, indicate whether each material, system or method listed is relevant to your study. If you are not sure if a list item applies to your research, read the appropriate section before selecting a response.

Materials & experimental systems

n/a	Involved in the study	
☐	☒	Antibodies
☐	☒	Eukaryotic cell lines
☒	☐	Palaeontology
☐	☒	Animals and other organisms
☒	☐	Human research participants
☒	☐	Clinical data

Methods

n/a	Involved in the study	
☒	☐	ChIP-seq
☒	☐	Flow cytometry
☒	☐	MRI-based neuroimaging

Antibodies

Antibodies used	Mouse Anti-6xHis antibody-horseradish peroxidase (HRP) (MA1-80218, Thermo Fisher Scientific), Mouse Anti-TFF3 primary antibody (WH0007033M1, Sigma), Mouse anti-LPS (1:200, Abcam ab35654), Rabbit anti-Mucin-2 (Santa Cruz sc-15334) and Goat anti-E-cadherin (1:200, R&D Systems AF648)
Validation	For MA1-80218, Western Blot (WB), ELISA, For WH0007033M1, Immunohistochemistry (IHC-P), WB, ELISA, For ab35654, IHC-Fr, ICC/IF, IHC-P, IP, ELISA, WB, For sc-15334, IF, IHC-P, For AF648, WB, Flow cytometry, ICC, IHC-P, Cy-TOF

Eukaryotic cell lines

Policy information about cell lines

Cell line source(s)	C2BBe1 [clone of Caco-2] (ATCC® CRL2102™)
Authentication	Cell lines were not authenticated
Mycoplasma contamination	Cell lines were not tested for mycoplasma contamination
Commonly misidentified lines<br>(See ICLAC register)	No commonly misidentified lines for Caco-2 cells in ICLAC

Animals and other organisms

Policy information about studies involving animals; ARRIVE guidelines recommended for reporting animal research

Laboratory animals	Female 8- to 9-week-old C57BL/6NCrl mice (mus musculus) were used

Wild animals	The study did not involve wild animals.
Field-collected samples	The study did not involve field-collected samples
Ethics oversight	the Harvard Medical Area Standing Committee on Animals (HMA IACUC)

Note that full information on the approval of the study protocol must also be provided in the manuscript.
